# “Get the Consent”—Nonfinancial Conflict of Interest in Academic Clinical Research

**DOI:** 10.1200/JCO.2016.69.3655

**Published:** 2016-10-03

**Authors:** Abby R. Rosenberg

**Affiliations:** Abby R. Rosenberg, Seattle Children’s Hospital and University of Washington School of Medicine, Seattle, WA.

During a presentation at the 2016 ASCO Annual Meeting in Chicago, Illinois, a bioethicist joked that financial conflict of interest (COI) disclosures were the great success of modern bioethics.^1^ I appreciated the irony; perhaps our focus on financial COI has overshadowed other competing interests at the intersection of academic medicine and clinical research. Nonfinancial COIs may be equally pervasive, are often unavoidable, and are no less important.^2-6^

## Perceptions and Missed Opportunities

First, it is important to underscore perceptions of COI. Whereas legal entities evaluate COI on the basis of divergent objective, regulatory, and/or structural rules, medical communities associate it with fraudulent, even overtly unethical, circumstances.^7^ This is probably a result of highly publicized historical scandals involving investigators receiving monetary gains from sponsoring pharmaceutical companies,^8-13^ inaccurate publication of results on the basis of financial (dis)-incentives,^14-16^ and evidence suggesting that pharmaceutical sponsorship in medical education induces biases.^15,17-21^ For these reasons, ASCO and other organizations, including the National Institutes of Health, the US Food and Drug Administration, and the Institute of Medicine, have all made the disclosure of financial COI mandatory.^13,22-26^

None of these organizations have explicitly recognized nonfinancial COI, however. This is a problem because completely separating physician-investigators’ competing interests in patient care, goals for scientific advancement, and individual and institutional ambitions for leadership and accomplishment is unrealistic, if not untenable. Indeed, professional job security and advancement for academic physicians is often contingent on successful competition for extramural funding and/or publication of scholarly work, both of which require fruitful research endeavors. In the case of research that involves human subjects and clinical trials, this means enrolling patients.^13^ Ignoring these legitimate competing interests is a missed opportunity.

## Personal Examples

Although there are myriad examples where our academic and clinical interests may compete, here I underscore two instances related to informed-consent processes. Both relate to the influential, and perhaps unrecognized, biases created when professional success is tied to patient enrollment. First, as a pediatric oncology fellow, I was often tasked with getting the consent from a new patient with cancer. Like many of us, I was trained in the ethical conduct of research and processes of informed consent. I could recite the relevant regulations and bioethical principles. I knew the evidence regarding coercion and (financial) COI. I had come to understand the value of clinical research in improving clinical oncology outcomes. I had been present for a handful of supervisor-led consent conferences. Now, the responsibility of leading the conference, and presumably enrolling the patient in the clinical trial, fell to me.

Here is the conundrum. After more than a decade of formal medical training, I had come to perceive that my supervisors’ approval, and hence my potential for continued academic achievement, was contingent on completed tasks and checklists. I heard “get the consent” and walked into the patient’s room with a clear agenda: consent equaled success; dissent equaled failure. In all likelihood, my task-oriented role colored my approach and ultimate impression of proficiencies.

Little empirical research has been conducted regarding trainees’ perceived COI in obtaining a patient’s informed consent; this example is entirely anecdotal. However, there is relevant precedent when trainees are asked to obtain do-not-resuscitate (DNR) orders.^27^ Here, task-oriented rather than process-oriented approaches (eg, “getting the DNR” rather than “beginning to explore family preferences and goals of care”) may be associated with a failure to meet the overarching best interests of the patient. For example, in a 1985 study of the use of DNR orders at three teaching hospitals, investigators found that discussions of resuscitation frequently failed to incorporate patient and family values; DNR orders were not fulfilling their goals.^28^ This has not changed in decades. Current literature highlights persistent problems with DNR processes, including the fact that trainees receive inadequate skills training and role modeling to appropriately conduct these conversations.^29^ Many have called for a reform of DNR policies and hospital culture to optimize bioethical principles of autonomy, beneficence, nonmaleficence, and justice.^30,31^

Returning to my own experience, during my fellowship I did not yet understand that if a family said “no” to a study, it was not a marker of personal failure or that successful consent conferences could have a variety of outcomes. Instead, I grappled with multiple, potentially conflicting interests: one to the patient, one to myself as a successful trainee, and perhaps an additional one to my institution’s reputation.

Indeed, regardless of physician-level success in obtaining informed consent, not enrolling a patient may still be associated with institutional failure. To encourage enrollment, nearly three-quarters of research sites are compensated by sponsor-led, so-called competitive enrollment practices where, for example, sites that recruit a sufficient number of participants are rewarded with additional enrollment slots (thereby receiving more ideal authorship positions on manuscripts or higher recognition).^13^ Sites that fail to meet enrollment quotas may be dropped from the study or the larger consortium. These incentives are highly likely to explicitly or implicitly bias investigators’ decisions and framing of consent conferences with potential participants; described hazards of competitive enrollment include inadequate disclosure of risks and exaggeration of potential benefits of study participation, enrollment of subjects who do not meet eligibility criteria, failure to report adverse events to oversight committees, improper data manipulation, and failure to terminate trials when indicated.^32^

Thus the quality of competing interests during informed consent has evolved with my career. Now an attending pediatric oncologist, I and my institutional colleagues are members of the Children’s Oncology Group (COG), the worlds’ largest cooperative clinical trial group for pediatric cancer. Within the COG, there is a team of investigators and select institutions tasked with conducting early (phase I) clinical trials. The selection of which trials to open within these sites is based on expert scientific opinion and extensive investigation of promising drugs and biologic agents.

As a member of the phase I consortium, my institution’s success in phase I patient enrollment is tracked. Indeed, if enrollment or other benchmarks fail to meet committee standards, then we may be placed on probation. If our numbers do not improve after a specified monitoring period, we may be removed from the program. Although the potential for COI is mitigated by the allowance of a 3-year rolling average, the pressure to successfully enroll a sufficient number of patients remains. We have an institutional reputation and role to maintain; hence, when patients are eligible for phase I clinical trials, we may implicitly or overtly encourage their participation.

In a report on the ethical conduct of clinical research involving children, the Institute of Medicine explicitly discouraged financially-based recruitment incentives because such systems of rewards could undermine the integrity of the study or the process of informed consent.^33^ Institutional pressures to recruit sufficient numbers of children may be similarly influential. However, the argument that sites must maintain a competitive practice of enrollment and research conduct also has merit. In early clinical research, for example, local experience with processes of adverse event monitoring, data collection, and, yes, consent processes, may mitigate some of the risk. My institution tracks not only successful recruitment, but also compliance with safety reporting and data-collection standards. Indeed, these latter standards are just as important to our inclusion in the consortium as sheer volume of enrollment. Likewise, as a result of the rigorous monitoring criteria, we have a dedicated team of investigators, nurses, and research associates who systematically and critically evaluate our research practices. In the end, the ethical and successful conduct of the research may be optimized.

## Conflict Resolution

Others have described opportunities to improve the content and process of informed consent conferences, including communication skills training.^34-37^ To my knowledge, none have explicitly addressed the role or effect of nonfinancial COI in these settings. How, then, do we proceed? Common sources of guidance include local, regional, and national policies.^12^ These are only part of the solution. In addition, there must be a standardized and nonthreatening opportunity for discussion of institutional and individual values, norms, and practices.^38^ These should include educational expectations and performance metrics (eg, you will not fail just because the family said “no”), thoughtful evaluations of enrollment practices within consortia (eg, how best can we optimize the safe, successful, and ethical conduct of phase I research?), as well as formal and directed training in the navigation and open discussion of competing interests (eg, how can we raise awareness and help with the navigation of nonfinancial COI?).

Unfortunately, such efforts may not fully mitigate the problem. For example, the Institute of Medicine has acknowledged that simple disclosure of COI may be ineffective, at least when the COI is financially mediated.^12^ The reasons are as follows. First, the value-laden response to reported financial COI creates a new, nonfinancial conflict; namely, investigators may feel shamed or discredited with the disclosure itself. Hence, there may be an incentive for nontransparency, or even dishonesty.^2^ Second, disclosing the conflict does not magically erase it. There is no clear evidence that disclosure requirements make investigators less likely to engage in monetary relationships with industry, nor do disclosures necessarily have an impact on the actual conduct of the research.^39^

Alternatively, research studies in the related field of unconscious bias suggest that focused training, personal awareness, and faculty role modeling can successfully shape behaviors and minimize implicit racial and weight biases.^40-43^ Perhaps we can translate these findings to nonfinancial COI. For example, trainees who witness successful consent conferences that do not end in enrollment may feel more equipped to realistically assess their own competencies and communication styles.

We also must conduct our own empirical research. As with the regulations, the limited studies to date have focused on financial as opposed to nonfinancial COI. A survey of physicians found that 52% believed that their colleagues were likely to be biased by industry-sponsored gifts, even of nominal value, whereas only 36% believed themselves to be similarly at risk.^20^ Among potential research participants, another survey-based study found that willingness to participate in clinical trials was strongly influenced by disclosed financial COI; 64% of respondents said that knowing about financial conflicts was extremely or very important, and many said that they would be reluctant to enroll if such conflicts were present.^44^

There is no similar research describing the effect of nonfinancial COI on research outcomes or the well-being of physicians and patients. Early studies might ask how institutional enrollment expectations affect individual provider styles during consent conferences, or if there are systematic differences in procedures between centers that are and that are not members of cooperative consortia. As we have done with financial COI, investigations might also evaluate patient decision making in the context of nonfinancial COI. At a minimum, rigorous investigation of the prevalence and influence of nonfinancial COI is necessary if we hope to successfully navigate it.

COIs (financial and nonfinancial) also need to not be labeled as unethical. As with the example of the COG phase I consortium, there is potential for both harm (undue influence to obtain a consent) and benefit (safer and more experienced conduct of research that involves human subjects). What matters most is that individuals and institutions have effective tools to manage COI when they arise. What these tools look like, and how they are implemented, must be tailored to individuals, institutions, and perhaps specific circumstances.^3,45^

Finally, we must accept the fact that nonfinancial COIs are pervasive and unavoidable in clinical medicine. They are rarely corrupt and include the examples here, as well as topics of professional publication practices and pressures, dual responsibilities of education and bedside care, and more general bioethical debates. For example, clinical ethics consults often arise when providers have competing interests (eg, the allocation of scare resources), when tensions exist between patient autonomy and beneficence, or when the provider must navigate shared roles of family members in decision making. In these cases, we approach the problem solving with methodological frameworks and systematic discussion. Let this practice be translated into all settings of conflicting commitments.

## References

[1] Caplan A

[2] Brody H (2011). Clarifying conflict of interest. Am J Bioeth.

[3] Saver RS (2012). Is it really all about the money? Reconsidering non-financial interests in medical research. J Law Med Ethics.

[4] Levinsky NG (2002). Nonfinancial conflicts of interest in research. N Engl J Med.

[5] Korn D (2001). Conflicts of interest. Science.

[6] Horrobin DF (1999). Beyond conflict of interest. Non-financial conflicts of interest are more serious than financial conflicts. BMJ.

[7] Johnson SH (2004). Five easy pieces: Motifs of health law. Health Matrix Clevel.

[8] Rosenbaum L (2015). Conflicts of interest: Part 1. Reconnecting the dots—Reinterpreting industry-physician relations. N Engl J Med.

[9] Goldner JA (2000). Dealing with conflicts of interest in biomedical research: IRB oversight as the next best solution to the abolitionist approach. J Law Med Ethics.

[10] Wilson RF (2010). The death of Jesse Gelsinger: New evidence of the influence of money and prestige in human research. Am J Law Med.

[11] Goldner JA (2009). Regulating conflicts of interest in research: The paper tiger needs real teeth. St Louis Univ Law J.

[12] Lo B, Field MJ (2009). Conflicts of Interest in Medicine, Research, Education, and Practice.

[13] Coleman CH, Menikoff JA, Goldner JA (2015). The Ethics and Regulation of Research with Human Subjects.

[14] Blumenthal D, Campbell EG, Anderson MS (1997). Withholding research results in academic life science: Evidence from a national survey of faculty. JAMA.

[15] Bekelman JE, Li Y, Gross CP (2003). Scope and impact of financial conflicts of interest in biomedical research: A systematic review. JAMA.

[16] Smith L, Byers JF (2002). Gene therapy in the post-Gelsinger era. JONAS Healthc Law Ethics Regul.

[17] Chren MM, Landefeld CS (1994). Physicians’ behavior and their interactions with drug companies. A controlled study of physicians who requested additions to a hospital drug formulary. JAMA.

[18] Cain DM, Loewenstein G, Moore DA (2005). The dirt on coming clean: Perverse effects of disclosing conflicts of interest. J Legal Stud.

[19] Boyd EA, Cho MK, Bero LA (2003). Financial conflict-of-interest policies in clinical research: Issues for clinical investigators. Acad Med.

[20] Korenstein D, Keyhani S, Ross JS (2010). Physician attitudes toward industry: A view across the specialties. Arch Surg.

[21] Greenwood K, Coleman CH, Boozang KM (2012). Toward evidence-based conflicts of interest training for physician-investigators. J Law Med Ethics.

[22] American Society of Clinical Oncology (2013). American Society of Clinical Oncology: Policy for relationships with companies. J Clin Oncol.

[23] Borus JF, Alexander EK, Bierer BE (2015). The Education Review Board: A mechanism for managing potential conflicts of interest in medical education. Acad Med.

[24] Sierles FS, Kessler KH, Mintz M (2015). Changes in medical students’ exposure to and attitudes about drug company interactions from 2003 to 2012: A multi-institutional follow-up survey. Acad Med.

[25] National Institutes of Health Financial Conflict of Interest. http://grants.nih.gov/grants/policy/coi/.

[26] National Institutes of Health Conflict of Interest. https://ethics.od.nih.gov/Topics/coi.htm.

[27] Tulsky JA, Chesney MA, Lo B (1996). See one, do one, teach one? House staff experience discussing do-not-resuscitate orders. Arch Intern Med.

[28] Evans AL, Brody BA (1985). The do-not-resuscitate order in teaching hospitals. JAMA.

[29] Yuen JK, Reid MC, Fetters MD (2011). Hospital do-not-resuscitate orders: Why they have failed and how to fix them. J Gen Intern Med.

[30] Weissman D (2003). Policy proposal: Do not resuscitate orders: A call for reform. Virtual Mentor.

[31] Dzeng E, Colaianni A, Roland M (2015). Influence of institutional culture and policies on do-not-resuscitate decision making at the end of life. JAMA Intern Med.

[32] Barnes M, Florencio PS (2002). Financial conflicts of interest in human subjects research: The problem of institutional conflicts. J Law Med Ethics.

[33] Field MJ, Berhman RE (2004). Ethical Conduct of Clinical Research Involving Children.

[34] Jenkins V, Solis-Trapala I, Langridge C (2011). What oncologists believe they said and what patients believe they heard: An analysis of phase I trial discussions. J Clin Oncol.

[35] Fallowfield LJ, Solis-Trapala I, Jenkins VA (2012). Evaluation of an educational program to improve communication with patients about early-phase trial participation. Oncologist.

[36] Butow P, Brown R, Aldridge J (2015). Can consultation skills training change doctors’ behaviour to increase involvement of patients in making decisions about standard treatment and clinical trials: A randomized controlled trial. Health Expect.

[37] Brown RF, Butow PN, Boyle F (2007). Seeking informed consent to cancer clinical trials: Evaluating the efficacy of doctor communication skills training. Psychooncology.

[38] Ferguson K, Masur S, Olson L (2007). Enhancing the culture of research ethics on university campuses. J Acad Ethics.

[39] Williams-Jones B (2011). Beyond a pejorative understanding of conflict of interest. Am J Bioeth.

[40] Stone J, Moskowitz GB (2011). Non-conscious bias in medical decision making: What can be done to reduce it?. Med Educ.

[41] Byrne A, Tanesini A (2015). Instilling new habits: Addressing implicit bias in healthcare professionals. Adv Health Sci Educ Theory Pract.

[42] van Ryn M, Hardeman R, Phelan SM (2015). Medical school experiences associated with change in implicit racial bias among 3547 students: A Medical Student CHANGES study report. J Gen Intern Med.

[43] Phelan SM, Puhl RM, Burke SE (2015). The mixed impact of medical school on medical students’ implicit and explicit weight bias. Med Educ.

[44] Kim SY, Millard RW, Nisbet P (2004). Potential research participants’ views regarding researcher and institutional financial conflicts of interest. J Med Ethics.

[45] Davis M, Stark A (2001). Conflict of Interest in the Professions.

